# Antifungal Activity of Endophytic *Bacillus* K1 Against *Botrytis cinerea*

**DOI:** 10.3389/fmicb.2022.935675

**Published:** 2022-07-22

**Authors:** Peiqian Li, Baozhen Feng, Zhen Yao, Bohui Wei, Yanfei Zhao, Shouguo Shi

**Affiliations:** Key Laboratory of Plant Disease and Pest Control, Life and Science Department, Yuncheng University, Yuncheng, China

**Keywords:** grape, endophyte, *Bacillus*, antifungal activity, VOCs, biosynthetic gene clusters

## Abstract

Gray mold caused by *Botrytis cinerea* is detrimental to plants and fruits. Endophytes have been shown to modify plant disease severity in functional assays. We conducted this study to investigate the endophytic strain *Bacillus* K1 with excellently antagonistic *B. cinerea* from the wild grape endosphere. We identified a wild grape endophytic strain K1 with high antifungal activity against *B. cinerea* both *in vitro* and *in vivo*. Combining the phylogenetic results based on 16S rDNA and genome sequencing, K1 was assigned as *Bacillus subtilis*. The *in vitro* results displayed that K1 and its volatile substances could significantly inhibit the mycelia growth of *B. cinerea*. Grape fruit inoculated with *Bacillus* K1 showed lower gray mold during treatment. The higher levels of defense-related enzymes, including peroxidase, polyphenol oxidase, and phenylalanine ammonia lyase, were induced in grapes after inoculation. Scanning electron microscopy (SEM) suggested that K1 inhibited mycelial growth *via* bacterial colonization and antibiosis in grapes. The gas chromatography–mass spectrometry analysis identified 33 volatiles in which dibutyl phthalate was the major compound accounting for 74.28%. Dibutyl phthalate demonstrated strong activity in suppressing the mycelia growth of *B. cinerea*. Genome bioinformatics analysis revealed that the K1 chromosome harbored many known biosynthesis gene clusters encoding subtilosin, bacillaene, bacillibactin, bacilysin, and fengycin. This study provides a potential biological agent to control diseases of post-harvest grape fruit and improves our understanding of the possible biocontrol mechanisms of the *Bacillus* strain.

## Introduction

Gray mold caused by *Botrytis cinerea* is one of the most severe diseases in post-harvest fruit and vegetables (Wang et al., 2021). It affects the quality and causes extensive damage to fruits and vegetables. Chemical control is still the main method to control post-harvest gray mold; however, the continuous use of fungicides can put great pressure on environmental safety and animal and human health, thus affecting the sustainable development of human society (Vikram et al., 2021).

Biocontrol with microbial antagonists in managing fruit post-harvest pathogens is a promising alternative treatment (Zhu et al., 2020). In recent years, many beneficial microbial agents, including *Bacillus halotolerans* against *B. cinerea* on strawberry fruit (Wang et al., 2021), *Bacillus amyloliquefaciens* against pathogens on loquat fruit (Ye et al., 2021), *Streptomyces* against *Colletotrichum fragariae* on strawberry fruit (Li et al., 2021), and *Metschnikowia pulcherrima* against *Penicillium expansum* on apples (Settier-Ramírez et al., 2022), have been identified as biological control agents for post-harvest plant diseases. Endophytic bacteria are considered an important biocontrol microbial agent in plant disease control (Afzal et al., 2019). They are beneficial microorganisms colonizing the internal tissues of plants and promoting plant growth without any harmful effects (Santoyo et al., 2016). Endophytic bacteria can benefit plants by assisting plant in nutrient acquisition and promoting plant growth by modulating phytohormones production (Vacheron et al., 2013; Ma et al., 2016). Additionally, endophytes are known to improve plant health *via* the inhibition of pathogens through the production of antibiotics, lytic enzymes, and volatile compounds (Bruisson et al., 2019; Omomowo and Babalola, 2019; Chaouachi et al., 2021).

Endophytic bacteria have been isolated from many agronomic crop plants and some wild plants. The most isolated bacterial genera are *Bacillus, Burkholderia, Microbacterium, Micrococcus, Pantoea*, and *Pseudomonas*, among which *Bacillus* and *Pseudomonas* are the predominant genera (Chaturvedi et al., 2016; Afzal et al., 2019). However, fewer studies have investigated the biocontrol effect of endophytic bacteria on gray mold disease of post-harvest grapes during storage.

We conducted this study to (1) screen and identify endophytic strain *Bacillus* K1 with excellently antagonistic *B. cinerea* from the wild grape endosphere, (2) evaluate the biocontrol effect of endophytic strain K1 against *B. cinerea* in grapes during storage, and (3) assess its antifungal mechanisms. The study results can provide more information on a promising bacterial biocontrol agent for controlling post-harvest gray mold.

## Materials and Methods

### Isolation of Endophytic Bacterial Strain

Wild grape plant (*Vitis heyneana* Roem. et Schult) samples were obtained from the primitive ecological area of Zhongtiao Mountains in Shanxi Province, China (latitude 35°09′N, longitude 111°20′E, and elevation 500 m). Endophytic bacterial strain K1 was isolated from plant materials by using a nutrient agar (NA) medium as previously described (Dobereiner et al., 1993). The isolated strain was maintained in 30% glycerol solution at −20°C.

### Primary Screening for Antagonistic Activity of K1 Against *Botrytis cinerea*

The endophytic strain K1 was streaked at the center of a Petri dish containing potato dextrose agar (PDA) medium. Following this, two 5-mm hyphal disks of *B. cinerea* cultures were manufactured and placed on both sides at an ~2.5-cm distance from the center. *B. cinerea* plates without bacteria were used as a control. Plates were incubated at 24°C in dark for 5–7 days. The growth diameters were tested when the control plates were fully covered with *B. cinerea* mycelia. The inhibition percentage was evaluated according to the method of Gao et al. (2017). Control Petri dishes contained only two mycelia disks of the fungal strains. Each treatment was tested in three independent replicates, and three Petri plates were used for each replicate. The experiment was repeated thrice.

### Secondary Screening of Antagonistic Activity of K1 Against *B. cinerea*

The K1 strain was incubated in 150-ml liquid Luria–Bertani (LB) broth at 30°C and 180 rpm for 24 h. The fermentation broth was collected and centrifuged at 4,000 × *g* for 10 min to collect K1 extract. Then, the extract was filtered through a 0.22-μm filter to collect K1 supernatant. Following this, the Oxford cup experiment was performed as follows: a 5-mm hyphal disk of *B. cinerea* was placed at the center of a PDA plate, then two Oxford cups were placed on each side at an ~2.5-cm distance from the pathogen, and 100 μl of K1 supernatant was added to the Oxford cups. PDA plate with a 5-mm hyphal disk of *B. cinerea* was used as the control. Plates were incubated at 24°C in the dark for 5–7 days. The inhibition percentage was calculated according to the method described above. The treatment was tested in three independent replicates, and three Petri plates were used for each replicate. The experiment was repeated thrice.

### Double-Plate Assay of Volatile Organic Compounds

A double-plate assay was used to study the antagonistic activity of volatile organic compounds (VOCs) against *B. cinerea* according to a previous study (Rajani et al., 2021). A 5-mm hyphal disk of *B. cinerea* culture was placed at the center of a PDA plate. A Petri plate having the inoculum of the bacterial strain K1 was inverted over the plate, and the plate was inoculated with the *B. cinerea* and incubated. Control treatment consisted of the mycelia disc of the pathogen alone. Plates were sealed with a cling wrap, and they were incubated at 24°C in the dark for 5–7 days. The inhibition percentage was calculated as described earlier. The treatment was performed in three independent replicates, and three Petri plates were used for each replicate. The experiment was repeated thrice.

### Analyses of VOCs *via* Gas Chromatography–Mass Spectrometry

The K1 strain was streaked in NA medium at 30°C for 10 h. A single colony was cultured in 100-ml LB liquid medium at 30°C and 200 rpm min^−1^ for 24 h. Following this, 20 μl of K1 suspension was transferred to 300 ml LB at 30°C and 200 rpm min^−1^ for 48 h. Later, the fermentation liquid was extracted with ethyl acetate (1:1) thrice, and the extraction liquid was concentrated *via* rotary evaporation at 35°C.

Subsequently, the strain extract was first dissolved in the chromatographic-grade methanol and filtered through a 0.2-μm filter. The solution was injected into a gas capillary column (DB-17MS, 30 m × 0.25 mm × 0.25 μm) of a gas chromatographer (5975C Inert XL MSD, Agilent, United States). Helium was used as the carrier gas with a flow rate of 1 ml min^−1^. The mass spectrometer electron ionization (EI) with a replaceable horn was operated in the EI mode at 70 eV with a continuous scan from 50 to 800 m/z. Peaks were identified by matching the mass spectra with the National Institute of Standards and Technology (NIST, United States) library.

### Genome Sequencing and Molecular Identification of K1 Strain

Genomic DNA of K1 was extracted using a bacterial genomic DNA isolation kit (Biotech Corporation, Beijing, China). The harvested DNA was detected *via* the agarose gel electrophoresis, and it was quantified using a Qubit^®^ 2.0 Fluorometer (Thermo Scientific, USA). 16S rRNA was amplified using the universal primers 27F and 1492R as previously described (Gao et al., 2017). The 16S rRNA sequence was blasted at NCBI (https://www.ncbi.nlm.nih.gov), and sequences of related strains were downloaded. A phylogenetic tree was then built according to the neighbor-joining method using Mega-X software (Kumar et al., 2018). The neighbor-joining tree was constructed based on bootstrap values with 1,000 replications. To further distinguish the K1 strain, the whole genome of K1 was sequenced using the Nanopore PromethION platform and Illumina NovaSeq PE150 at the Beijing Novogene Bioinformatics Technology Co., Ltd.

A total of 5,378,358,199-bp paired end reads were generated. These reads were assembled using Unicycler v0.4.7 (https://github.com/rrwick/Unicycler, Wick et al., 2017). K1 genome sequences were uploaded to the Type Strain Genome Server (TYGS) for genome-based taxonomic classification (Meier-kolthoff and Göker, 2019). The genome sequences of strain K1 were submitted to NCBI and assigned an accession number (CP093546).

### Genomic Annotation of the *Bacillus* Strain K1

The open reading frames and genome annotation were predicted using GeneMarkS v4.17 (http://topaz.gatech.edu/GeneMark/, Besemer et al., 2001). Genes were annotated using the Clusters of Orthologous Groups (COGs) (Galperin et al., 2015), Gene Ontology (GO) (Ashburner et al., 2000), and Kyoto Encyclopedia of Genes and Genomes (KEGG) (Ogata et al., 1999). Meanwhile, we analyzed the secondary metabolism gene clusters with antiSMASH v4.0.2 software (Blin et al., 2017).

### *In vivo* Antifungal Activity Assays

Fresh fruits were used for *in vivo* antifungal activity against *B. cinerea*. Victoria grape (*Vitis vinifera* L.) and cherry tomato (*Lycopersicon esculentum* L.) were picked from local cultivation. Healthy fruit was free from physical injuries and homogeneous in size and maturity. Prior to the experiments, fruits were rinsed with tap water, disinfected with 70% ethanol for 1 min, rinsed twice with sterile water, and air-dried. A uniform 2–3 mm deep wound was made on the surface of the fruit with a cork borer (5 mm diameter).

For *in vivo* antifungal efficacy test, 20 μl of samples (*Bacillus* strain K1 fermentation broth at 10^8^ CFU ml^−1^, or K1 suspension, or K1 supernatant) was injected into each wound. The K1 fermentation broth was collected from 12-h-old inoculum, and it was centrifuged at 4,000 × *g* for 10 min to obtain primary supernatant. Following this, the obtained liquid was filtered through a 0.22-μm filter to collect cell-free supernatant of K1. The precipitate was resuspended in sterile saline to regulate the cell concentration to 10^8^ CFU ml^−1^ as K1 suspension. Then, 20 μl of 10^6^ conidia mL^−1^ *B. cinerea* conidium suspension was inoculated into each wound, and the fruit was placed at 25°C and 80% of RH for 7 days. Fruits treated with *B. cinerea* conidium suspension were positive control. Three independent replicates were conducted for each treatment, and 12 grapes or 10 cherry tomatoes were used for each replication. The experiment was conducted thrice. Lesion diameters were measured to evaluate the biocontrol efficacy. The incidence rate of the gray mold was calculated as the percentage of decay symptoms to compare the treatments.

### Determination of Defense-Related Enzyme Activity

Following inoculation with 20 μl *Bacillus* K1 suspension (1 × 10^8^ CFU ml^−1^), samples were collected for defense-related enzyme activity assay. Three kinds of enzymes including peroxidase (POD), polyphenol oxidase (PPO), and phenylalanine ammonia lyase (PAL) were tested. Grapes injected with 20 μl double-distilled water were used as the control.

For POD and PPO assays, the crude enzyme extract was obtained in accordance with a previous method (Devaiah et al., 2009). In terms of PAL, the fruit flesh was homogenized as previously described (Ye et al., 2021). The protein content of extracts was measured with the Bradford Protein Assay Kit from Beyotime Biotechnology (Shanghai, China) according to the standard manufacturer's protocol. The enzyme activity was presented as an increase in A min^−1^ per mg protein.

The peroxidase activity was assayed using guaiacol and absorbance increased at a 470-nm wavelength (Pasquariello et al., 2015). Subsequently, 100 μl of crude enzyme extract was inoculated with a guaiacol reaction mixture containing 50 mM potassium phosphate (pH 6.4), 0.3% guaiacol, and 0.3% H_2_O_2_. The PAL activity was analyzed by measuring the conversion of guaiacol into tetraguaiacol at 470 nm.

The polyphenol oxidase activity was determined using catechol as the substrate at 398 nm (Ma et al., 1992). The reaction buffer contained 50 μl of crude enzyme extract, 10 mM catechol, and 50 M sodium phosphate (pH 6.4). The PPO activity was assayed by measuring the increase in the absorbance of the reaction mixture at 398 nm.

In terms of the PAL activity, the reaction mixture containing 700 μl of crude enzyme extract was inoculated with 50 mM L-phenylalanine in 200 mM borate buffer of pH 8.8 at 37°C for 8 h. Later, 56 μl of 6 M HCl was added to terminate the reaction. The PAL activity was quantified by monitoring the formation of trans-cinnamic acid and the consequent increase in absorbance at 290 nm in the reaction buffer (Benkeblia, 2000). Each treatment was tested in three independent replicates. All experiments were repeated three times.

### Scanning Electron Microscopy

After inoculation with *B. cinerea* and *Bacillus* K1 suspension, grape samples were prepared for SEM. Generally, the fruit samples were first fixed with 2.5% glutaraldehyde at 4°C for 24 h, and they were then post-fixed with 1% osmic acid for 1 h. Later, samples were dehydrated through graded concentrations of ethanol (30, 50, 70, 80, 90, 95, and 100%) for 15 min each, and they were later transferred to the mixture of alcohol and iso-amyl acetate (v:v = 1:1) for ~30 min. Finally, samples were transferred to liquid CO_2_ in a Hitachi Model HCP-2 critical point dryer. The dehydrated specimens were coated with gold, and they were observed under an SEM (HITACHI S-4800, Japan). Three scans were taken at three different magnifications, respectively, for each treatment. Individual parameters are visible in the picture bar of each image.

### Statistical Analysis

All data were analyzed *via* analysis of variance (ANOVA) using the SPSS statistical package (version 22, SPSS Inc., Chicago, IL, United States). The *t*-test was applied to compare means between different subjects. Statistical differences between means were assessed at the level of *p* < 0.05.

## Results

### *In vitro* Activity of Endophytic Strain K1 Against *B. cinerea*

**Figure 1:**
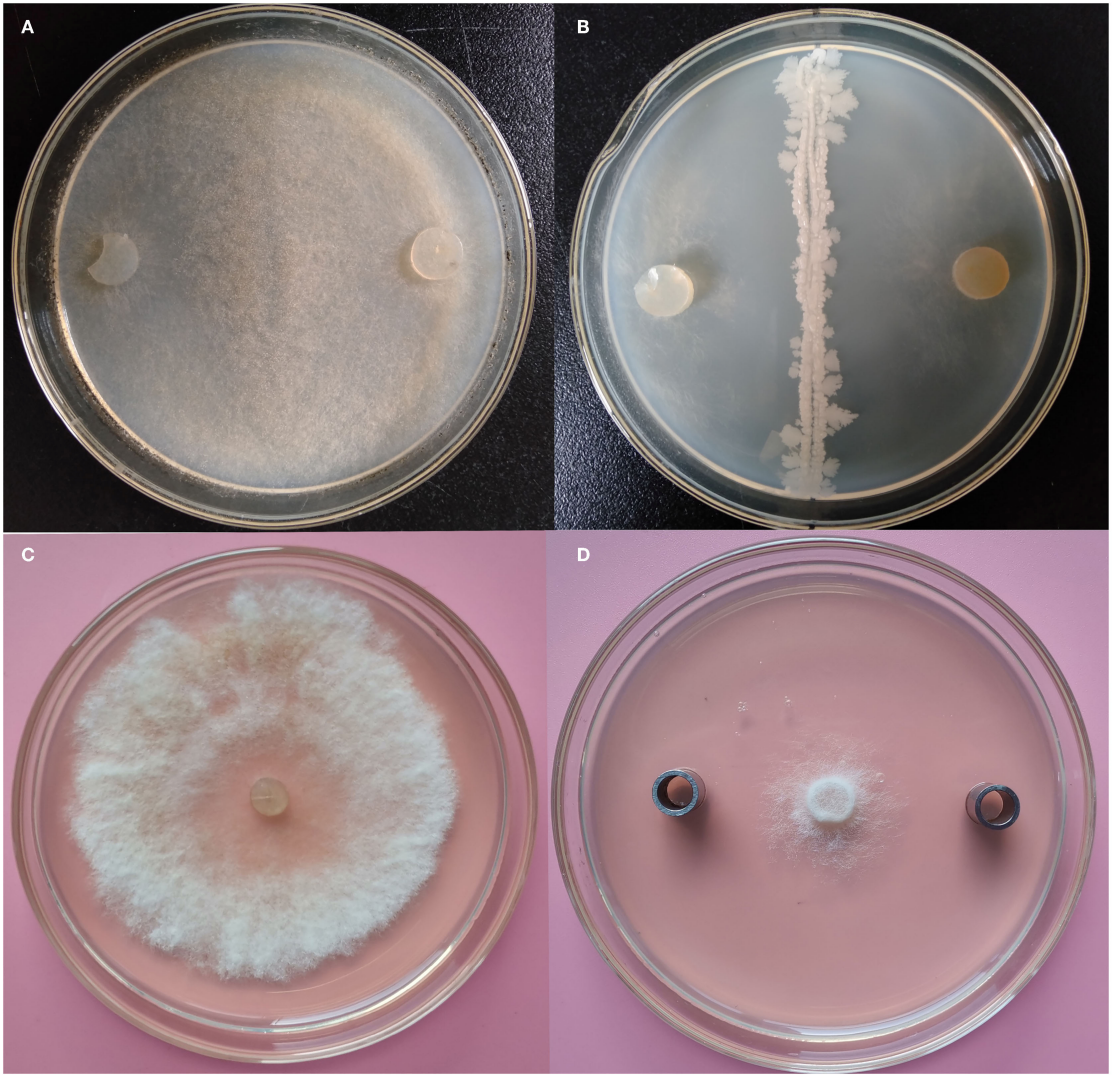
Antifungal evaluation of *Bacillus* K1 against *Botrytis cinerea*. **(A)** *B. cinerea* on potato dextrose agar (PDA) as control. **(B)** Growth inhibition of *B. cinerea* after antagonist with K1. **(C)** *B. cinerea* on PDA as control. **(D)** Growth inhibition of *B. cinerea* after antagonist with K1 extracts.

All isolated endophytic strains were subjected to a primary antifungal activity assay, and the strains showed different inhibition abilities on the mycelial growth of *B*. *cinerea*. Notably, the K1 strain showed the best antifungal activity, and the inhibition rate reached 78.42 ± 1.23% (Figures 1A,B; Supplementary Figure S1A).

The K1 strain was selected for small-scale fermentation in a 150-ml flask, and its antagonistic ability was further investigated. The results demonstrated that K1 extract had strong inhibition zone sizes, thus indicating its significantly antifungal activity against *B. cinerea* (Figures 1C,D). The inhibition rate of K1 was 85.46% ± 1.84% (Supplementary Figure S1B). Combining the antagonistic results, the K1 strain was selected as a potential biocontrol isolate for the subsequent studies.

### Antifungal Activity of VOCs and GC–MS Assay

**Figure 2:**
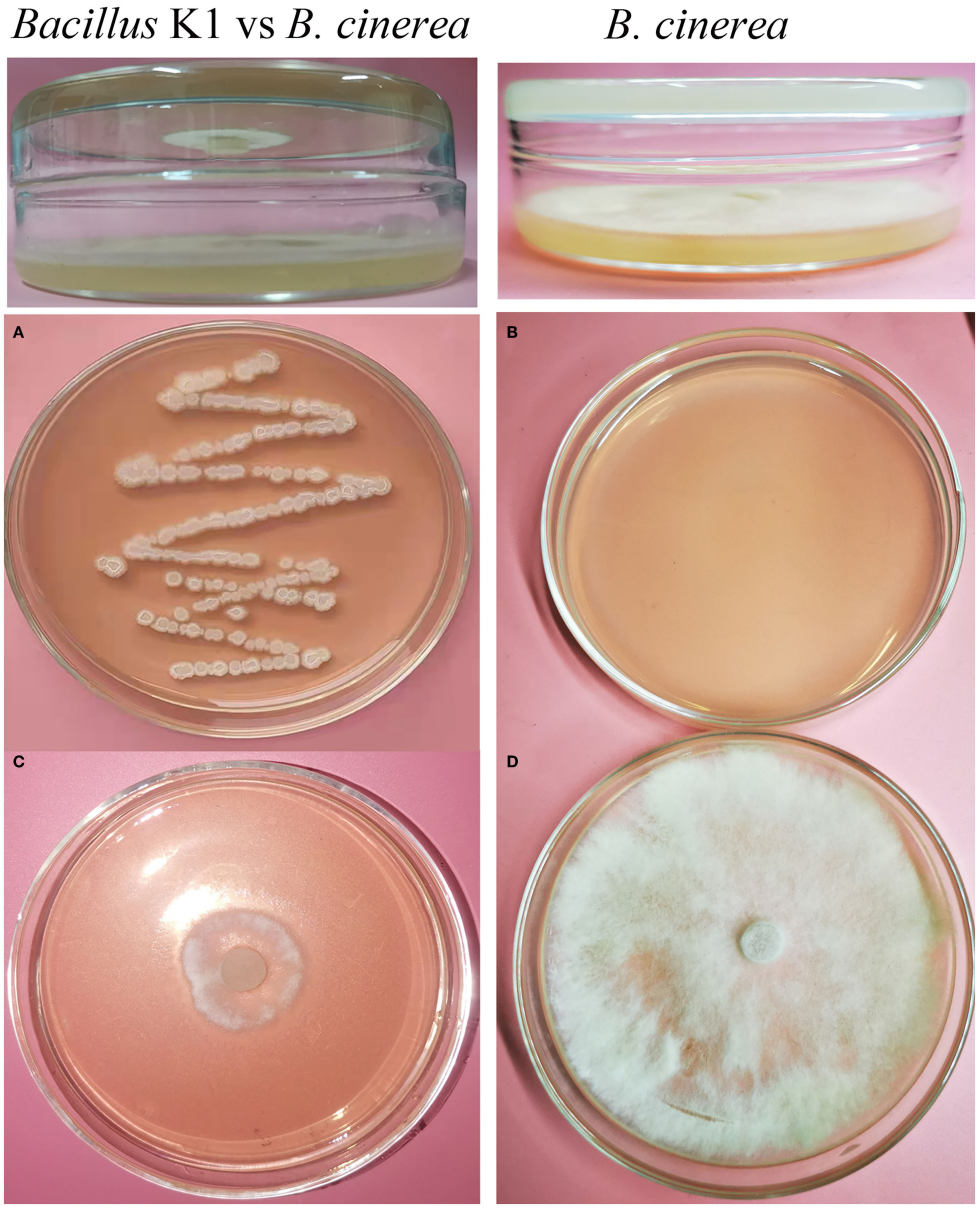
Inhibition of *Botrytis cinerea* by endophytic *Bacillus* strain K1 in double plate assay. **(A)** Endophytic *Bacillus* strain K1. **(B)** Blank plate as control (without endophyte). **(C)** Inhibition of *B. cinerea* by *Bacillus* strain K1. The petri plate with the bacterial strain K1 was inverted over the plate inoculated with the *B. cinerea* and incubated. **(D)** *B. cinerea* as control (without endophyte). In control, the blank petri plate was inverted over the *B. cinerea* plate.

A double plate assay was performed to test the antifungal activity of VOCs from K1. Our results demonstrated that *B. cinerea* growth was significantly inhibited by VOCs from the endophytic K1 strain. The inhibition percentage reached 83.32% ± 1.84% (as shown in Figure 2 and Supplementary Figure S1C), and it remained for 20 days from incubation.

In terms of GC–MS, a total of 33 compounds were identified based on a comparison of their mass spectra with the NIST library (Supplementary Table S1). The peak area represented the proportion of the given compound in the VOCs. According to the available library data, the main chemical compounds with an area percentage of more than 1% were identified as 3-methyl-butanoic acid, 2-methyl-butanoic acid, 3,5-dimethoxy-phenol, dibutyl phthalate, l-leucine-N-cyclopropylcarbonyl-hexadecyl-ester, [1,2-a]pyrazine-1,4-dione-hexahydro-3-(2-methylpropyl)-pyrrolo, (S-E)-2,3,7-trimethyl-4-octene, and (Z)-9-octadecenamide. Particularly, the results revealed a high content of dibutyl phthalate (74.28%) among all compounds.

### Identification of K1 Strain

**Figure 3:**
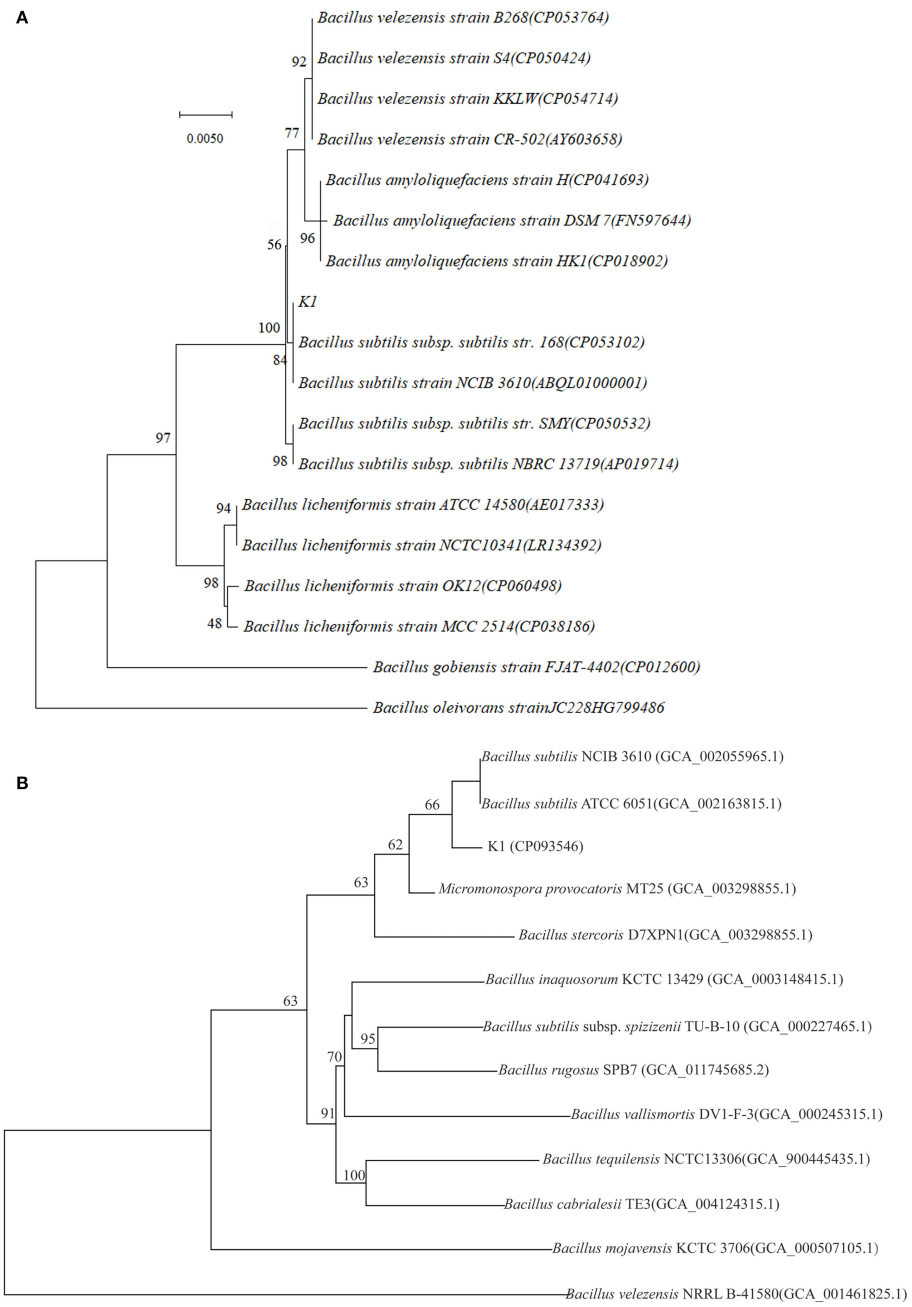
Identification of bacterial strain K1 based on 16S rRNA and Type Strain Genome Server (TYGS). **(A)** Phylogenetic analysis of K1 based on 16S rRNA sequences. **(B)** The minimum evolution tree based on the genome sequence of recognized strains from TYGS. The trees were constructed with bootstrap support based on 100 pseudo-bootstrap replicates.

The phylogenetic tree based on 16S rRNA sequences of K1 (1,468 bp, accession number: MW642497) and those of other *Bacillus* strains demonstrated that K1 was close to *Bacillus subtilis* strains (Figure 3A). To further identify the taxonomic affiliation of strain K1, its whole genome was sequenced and uploaded to TYGS for genome-based taxonomic classification. As shown in Figure 3B, K1 formed a distinct clade with *B. subtilis* in genome-based phylogenetic analyses (Figure 3B). Thus, our results suggested that K1 belonged to *B. subtilis*, and it was named after *Bacillus subtilis* K1.

### Bioinformatics Analysis of the Genome

**Figure 4:**
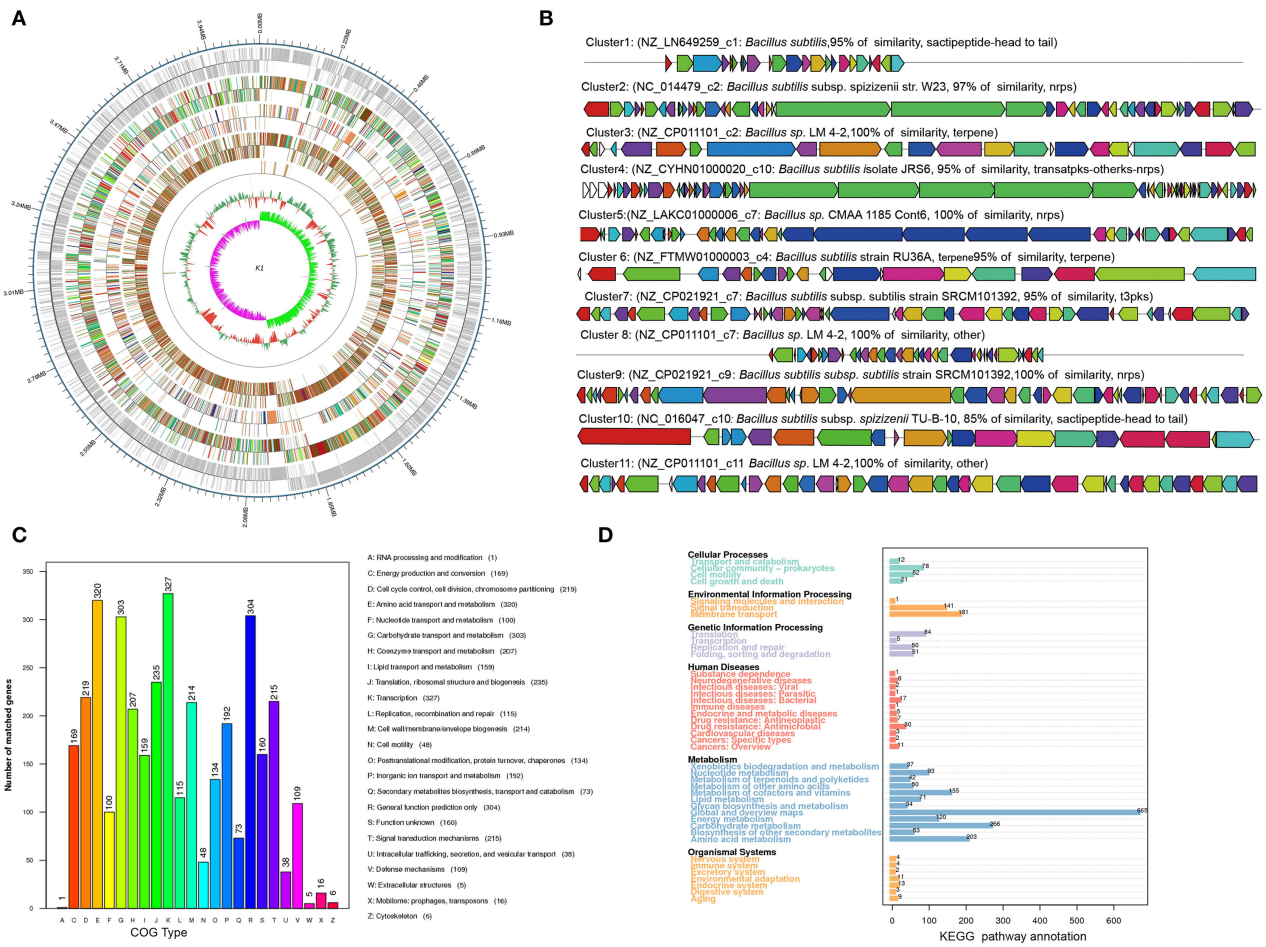
Genome information and function annotation of *Bacillus* K1. **(A)** Circular map of *Bacillus* K1 genome. From outside to center, ring 1 is the mark of genome size. Rings 2 and 3 represent coding genes on the forward/reverse strand. Rings 4 and 5 represent coding sequence (CDS) on the forward/reverse strand. Different colors indicate the functional category of different Clusters of Orthologous Groups (COGs) of CDS. Rings 6 and 7 represent CDS on the forward/reverse strand. Different colors indicate the functional category of different Kyoto Encyclopedia of Genes and Genomes (KEGG) pathway. Rings 8 and 9 represent CDS on the forward/reverse strand. Different colors indicate the functional category of different Gene Ontology (GO) function annotation. Ring 10 is tRNA and rRNA. Ring 11 shows the G + C content. The outward green part indicates that the guanine–cytosine (GC) content of this region is higher than the average GC content of the whole genome. The inward purple part indicates that the GC content of this region is lower than the average GC content of the whole genome. **(B)** Biosynthesis gene clusters demonstrating more than 90% similarity with the known sequences. **(C)** COG annotation of *Bacillus* K1 genome. **(D)** KEGG annotation of *Bacillus* K1 genome.

The whole *Bacillus* K1 genome contained 4,091,714 bp. The genome with 43.74% of the guanine–cytosine (GC) content included 86 tRNA genes and 4,263 coding sequences (CDSs) (Figure 4A). Using annotation, 86.07, 60.97, and 68.19% of CDSs were assigned to COG, KEGG, and GO, respectively. For 3,669 genes in COG, the top six categories contained transcription (327), amino acid transport and metabolism (320), general function prediction (304), carbohydrate transport and metabolism (303), translation, ribosomal structure, and biogenesis (235), and signal transduction mechanism (215) (Figure 4C). The KEGG annotation showed that 1,789 genes (68.83%) participated in the regulation of metabolism (Figure 4D). Using the antiSMASH software, 11 biosynthetic gene clusters were estimated in K1 genome sequences (Figure 4B; Table 1). The gene clusters included two head-to-tail sactipetides, two terpenes, one type III PKS, and one NRPS-beta-lactone. Genome analysis further revealed that seven biosynthetic gene clusters showed high similarity with subtilosin (100%), surfactin (80%), bacillaene (95%), fengycin (100%), bacillibactin (100%), and bacilysin (100%) (Table 1). The GO annotation showed that 2,907 genes were classified mainly into the biological process, cellular component, and molecular function (Supplementary Figure S2).

**Table 1:** Predicted gene clusters of biosynthesis in *Bacillus* K1.

**Clusters**	**Type**	**Initial position**	**Terminal position**	**Similar clusters**	**Similarity**	**Gene number**
Cluster1	Head_to_tail, sactipeptide	203,586	225,494	Subtilosin	100%	23
Cluster2	NRPS	359,499	422,332	Surfactin	80%	47
Cluster3	Terpene	1,133,711	1,154,224	–	–	24
Cluster4	NRPS, PKS-like, T3PKS, transAT-PKS, transAT-PKS-like	1,773,134	1,886,875	Bacillaene	95%	55
Cluster5	NRPS, betalactone	1,946,700	2,028,999	Fengycin	100%	42
Cluster6	Terpene	2,105,224	2,126,444	–	–	22
Cluster7	T3PKS	2,175,276	2,216,169	–	–	46
Cluster8	NRPS-like	2,619,814	2,661,135	Bacillaene	70%	37
Cluster9	NRPS	3,134,516	3,183,556	Bacillibactin	100%	45
Cluster10	Head_to_tail, sactipeptide	3,700,018	3,720,908	Subtilosin	100%	20
Cluster11	Other	3,725,675	3,764,951	Bacilysin	100%	43

### *In vivo* Inhibition Activity of *Bacillus* Strain K1 Against *B. cinerea* on Fruits

The lesion diameter on fruits was measured each day after the treatment, and photographs were taken to record the inhibition activity of K1 against gray mold caused by *B. cinerea*.

**Figure 5:**
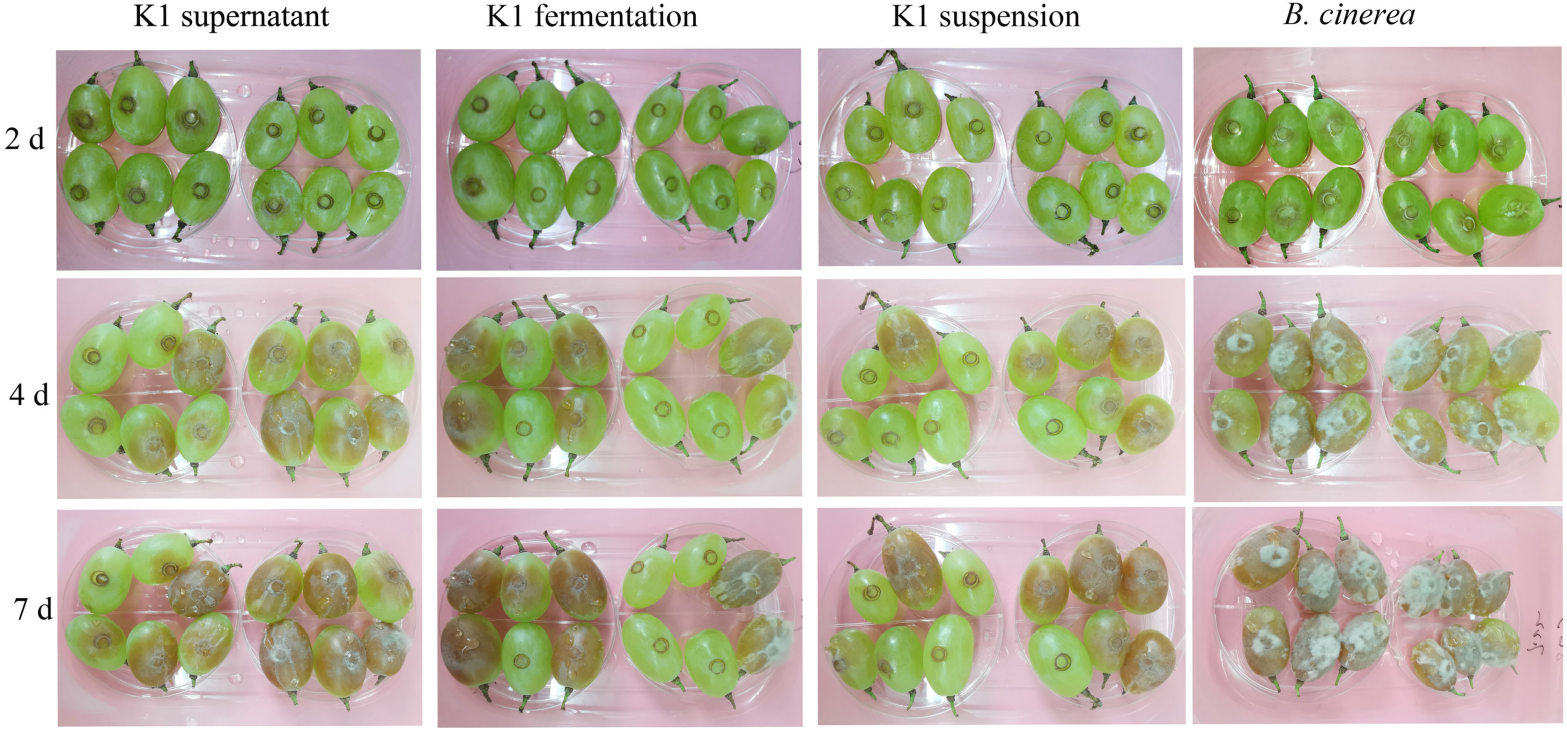
*In vivo* antifungal effect evaluation of K1 culture on grape fruit. Grapes were inoculated with K1 fermentation broth, supernatant, and suspension, respectively, and subsequently injected *B. cinerea* conidia suspension at the concentration of 10^6^. Control group were treated with *B. cinerea* conidia suspension (1 × 10^6^ ml^−1^).

**Figure 6:**
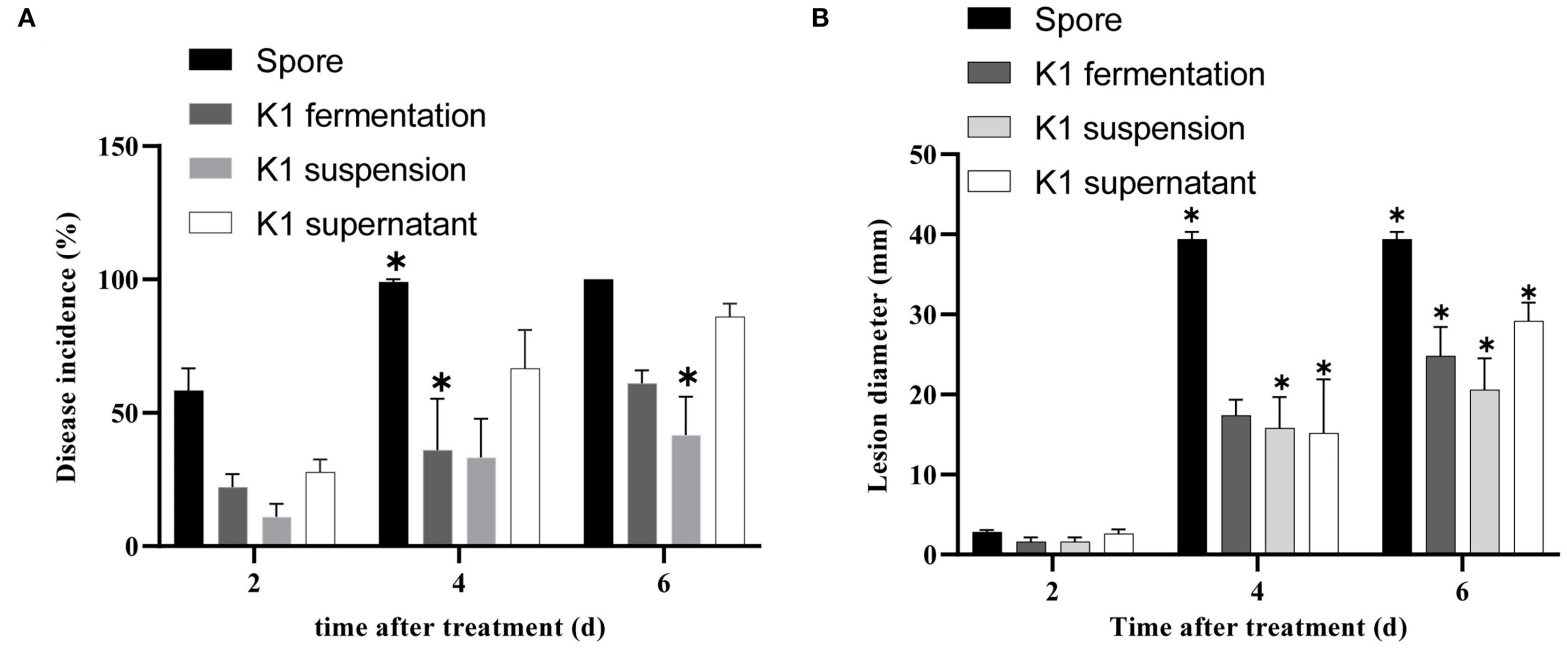
Efficacy of *Bacillus* K1 on grape fruit against *Botrytis cinerea in vivo*. **(A)** Disease incidence of gray mold on grape fruit after treatment with K1 fermentation, supernatant, and suspension. **(B)** Lesion diameter on grape fruit with different treatments. Three independent replicates were conducted for each treatment, 12 grapes were used for each replication. The experiment was conducted thrice. Values are means of three replicates. Error bars represent standard error of the mean, asterisks (*) represent significant differences according to a *t*-test (*p* < 0.05).

**Figure 7:**
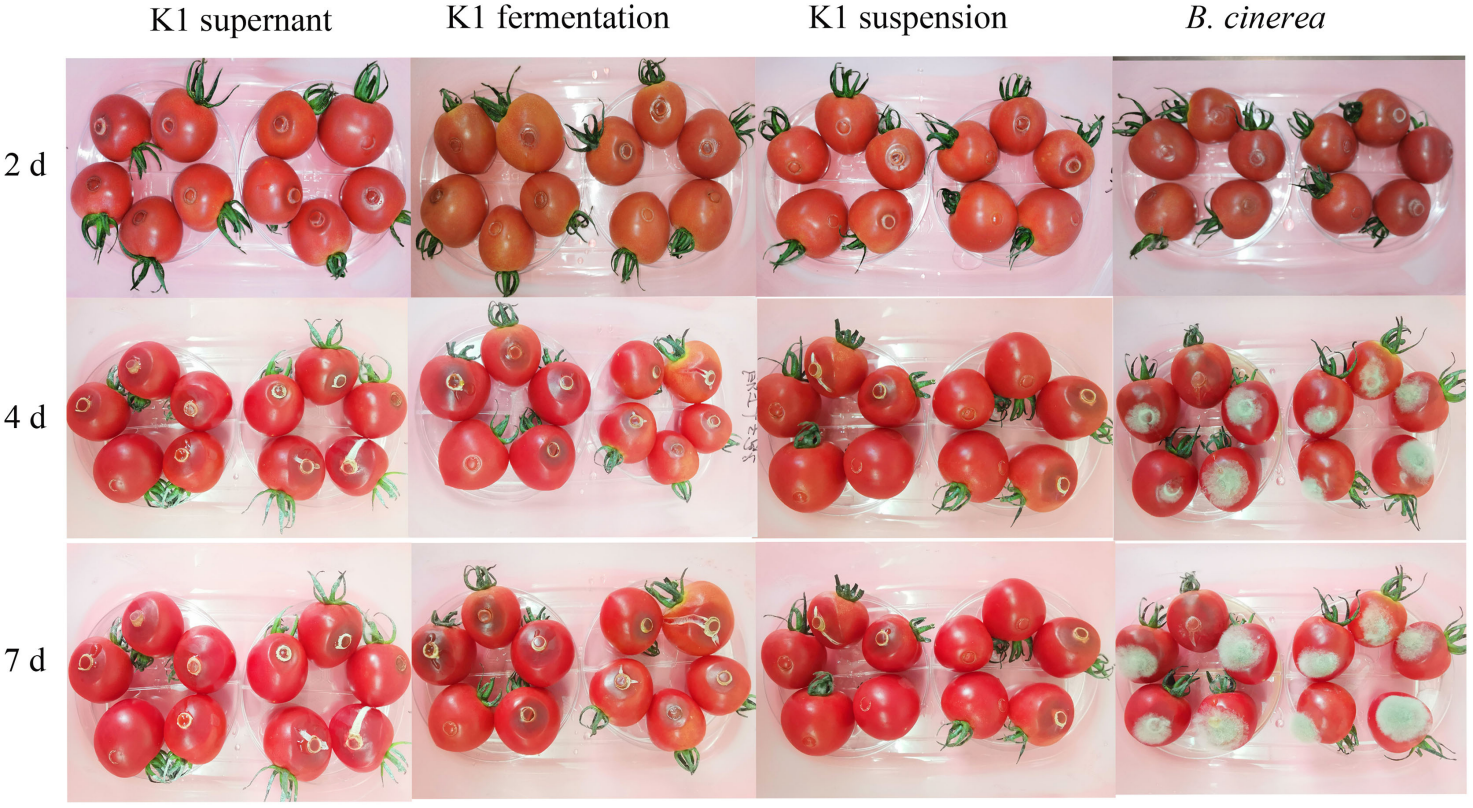
*In vivo* antifungal effect evaluation of K1 culture on tomato fruit. Tomatoes were inoculated with K1 fermentation broth, supernatant, and suspension, respectively, and subsequently injected *B. cinerea* conidia suspension at the concentration of 10^6^. Control group were treated with *B. cinerea* conidia suspension (1 × 10^6^ ml^−1^).

The K1 fermentation, supernatant, or suspension all had an obvious antifungal effect *in vivo* as compared to the control (Figures 5–**7**). Additionally, K1 suspension performed better than the others after inoculation with the pathogen for 6 days. The decay incidence of grape fruit groups inoculated with K1 was significantly lower than those of the *B. cinerea* group (Figure 5). The decay incidence of grape fruit in the conidium suspension group was 100%, whereas it was 40% in the suspension group (Figure 6A). After 6 days of inoculation, the lesion diameter of the treatment group with K1 suspension on grape fruit was only 18 mm, whereas that of the control group was 40 mm (Figure 6B). It was obvious that K1 could effectively delay the spoilage of grape fruit at a relatively high temperature.

In cherry tomato fruit treatment, *Bacillus* K1 presented an obvious antifungal activity during inoculation (Figure 7). As shown in Supplementary Figure S3, K1 treatment could significantly decrease fruit gray mold incidence rate. Cherry tomatoes inoculated with K1 suspension exhibited 48% of gray mold infection after 6 days, whereas the control exhibited 100% infection. Obviously, the lesion diameter of the treatment group with K1 suspension on grape fruit was only about 18 mm, whereas the control group was ~33 mm. Particularly, compared to control, all three constituents of K1 showed a lower incidence rate; however, K1 suspension had the best inhibition activity among the three treatments.

### Enzyme Activity Assay of Grapes Inoculated With K1 Suspension and *B. cinerea*

The PAL, POD, and PPO were defense-related enzymes in plant tissues. As shown in Figure 7, the activity of these three kinds of enzymes presented a dynamic change during the inoculation stage.

**Figure 8:**
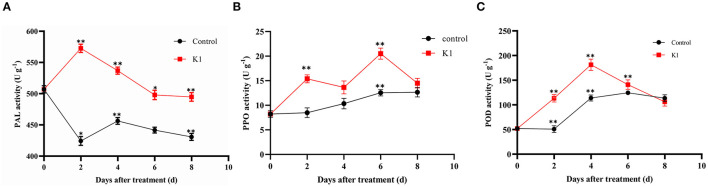
**(A–C)** Effects of K1 suspension on defense related enzyme activities of grapes. Grapes were treated with K1 suspension at the concentration of 10^8^ CFU ml^−1^. Grapes inoculated with 20 μl double distilled water as control. Error bars represent standard error of the mean. Statistical significance was determined according to independent sample *t*-test. * and ** indicate significant differences at *p* < 0.05 and *p* < 0.01, respectively.

The PAL activity significantly increased 2 days after treatment in the K1 suspension group, with up to 570 U g^−1^ of protein (Figure 8A). Additionally, the PAL activity of the K1 suspension group was higher than that of the control group during the test period, indicating that K1 suspension treatment could induce resistance in grapes to a certain degree.

The polyphenol oxidase activity of the K1 group rose to 15 U g^−1^ of protein on the second day, fluctuated to lower on the fourth day, and then gradually increased to a higher level of 20 U g^−1^ of protein toward the end (Figure 8B).

The peroxidase activity in treated grape fruit increased gradually, reached a peak of 190 U g^−1^ of protein on the fourth day, and then decreased to the same level as that of control in the end (Figure 8C).

### Scanning Electron Microscopy

**Figure 9:**
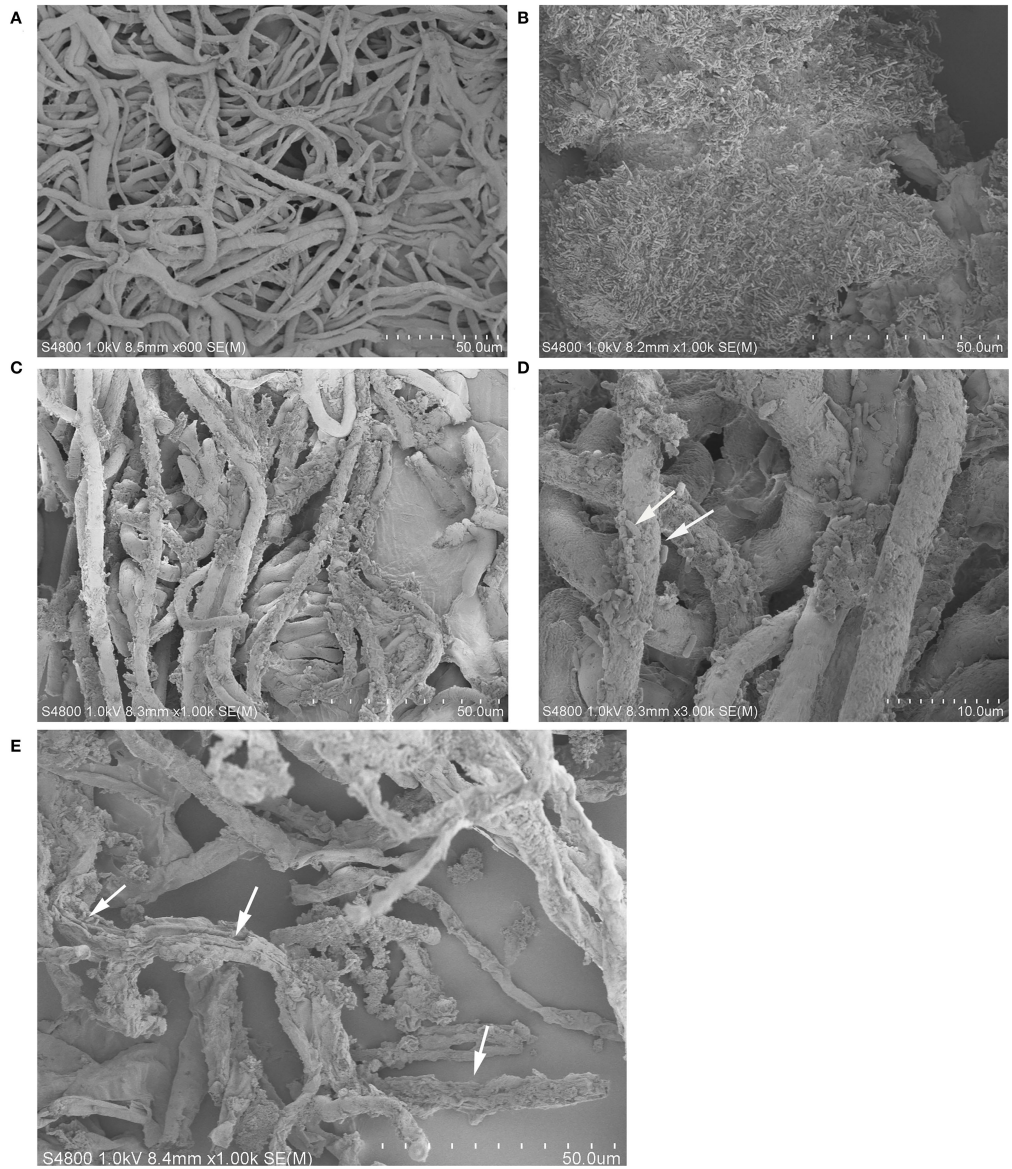
Interaction between endophytic *Bacillus* K1 and *Botrytis cinerea* in grape fruit. **(A)** General state of *B. cinerea* mycelia on grape after inoculation for 2 days. **(B)** Colonization of endophytic bacteria in grape tissue after inoculation with K1 suspension for 2 days. **(C,D)** Endophytic bacteria K1 attached to mycelia surface at ×1,000 and ×3,000 by scanning electron microscopy, respectively. The white arrows highlighted the cells of *Bacillus* K1. **(E)** Irreversible deformation and disruption of mycelia at 7 days after treatment indicated active antifungal substance released by *Bacillus* K1. The white arrows indicated the K1 cells and pores on mycelia.

Compared to pathogen treatment groups, grape fruit treated with K1 suspension was relatively smoother and more compact, indicating less pathogen activity. As seen in Figure 9, *Bacillus* K1 could colonize well in grape tissues and adjust the interaction with *B. cinerea* to achieve a better antifungal effect. The mycelia of the control group were regular, smooth, and densely interlaced (Figure 9A). In the presence of K1 suspension, many bacteria were attached to the mycelia surface (Figures 9C,D). The mycelium morphology was seriously deformed, showing irregular depressions and pores, and many secretions appeared on the surface on the seventh day after treatment (Figure 9E). The results indicated that active competition and antibiosis might be potential K1 antifungal mechanisms.

## Discussion

Grape (*Vitis vinifera* L.) is one of the most popular fruits in the world. However, it has a high susceptibility to microbial decay during the storage process. Gray mold caused by *B. cinerea* is the main pathogen affecting the quality of many fruits. Presently, modified atmosphere fumigation with SO_2_ is the main method for grape anti-corrosion storage. However, exogenously supplied SO_2_ causes cytoplasm acidification and radical formation, which might migrate to fruits and cause undesirable effects on food safety (Randewig et al., 2012). Exploring potential biological strains is considered an alternative strategy to solve these problems. In recent years, several studies have investigated biocontrol agents to inhibit plant pathogens. *B. subtilis* CU12 strain showed strong *in vitro* antifungal effects on *B. cinerea, Alternaria solani, Pythium sulcatum*, and *Fusarium sambucinum* (Wise et al., 2012). *B. amyloliquefaciens* strain BUZ-14 significantly decreased the mycelial growth of *Monilinia laxa* on fruits (Calvo et al., 2017). Strains *B. subtilis* KATMIRA1933 and *B. amyloliquefaciens* B-1895 have been employed in commercial production (Algburi et al., 2016), whereas *B. amyloliquefaciens* B4 shows excellent antifungal activity against *P. expansum* for post-harvest loquat fruit storage (Ye et al., 2021). *B. halotolerans* KLBC XJ-5 secretes the lytic enzyme chitinase and controls *B. cinerea* growth on post-harvest strawberries (Wang et al., 2021). In the present study, we identified a wild grape endophytic bacteria strain *B. subtilis* K1 based on 16S rRNA and whole genome-based analyses. The strain presented high antifungal activity on *B. cinerea* mycelia growth on Petri dishes. Additionally, the extract of K1 fermentation effectively suppressed *B. cinerea* mycelia growth (Figure 1). These results indicated that K1 could secrete some antibiotic compounds in fermentation, similar to previous studies (Wang et al., 2021; Ye et al., 2021). Moreover, K1 produced a set of antifungal VOCs using double plates assay (Figure 2). Several bacterial strains produced VOCs, and strain *B. velezensis* ZSY-1 exhibited significant antifungal activity against *Alternaria solani* and *B. cinerea* (Gao et al., 2017). Additionally, tomato endophytic bacteria, *B. nakamurai, B. pseudomycoides, B. proteolyticus, B. thuringiensis, Enterobacter asburiae*, and *E. cloacae* could produce bioactive compounds against *B. cinerea* (Chaouachi et al., 2021). Previous studies also highlighted the potential of bacterial VOCs to be used in crop protection in the field and storage environments (Arrarte et al., 2017; Dhouib et al., 2019; Zheng et al., 2019; Calvo et al., 2020).

The GS–MS assay indicated that K1 might emit 33 kinds of volatile compounds with dibutyl phthalate as the major constituent. Dibutyl phthalate is an antimicrobial bioactive compound produced by marine *Pseudomonas* strains (Hoang et al., 2008; Isnansetyo and Kamei, 2009). In *Streptomyces* strain BITDG-11, dibutyl phthalate demonstrates a strong antifungal activity against *Fusarium oxysporum* f. sp. *cubense* (Zhang et al., 2021). Furthermore, dibutyl phthalate has been reported as an antifungal compound in other actinomycete strains (Roy et al., 2006; Ahsan et al., 2017). To the best of our knowledge, this is the first study reporting the antifungal activity of dibutyl phthalate produced by *Bacillus*. Generally, endophytic bacterial strains produced bioactive VOCs along with different strain-specific and known antifungal VOCs such as 3-methylbutan-1-ol, pentadecane, 2-furanmethanol, 2-octanone, 2-heptanone, and dodecanal (Lawal et al., 2018; Calvo et al., 2020; Chaouachi et al., 2021).

*Bacillus* species have been reported to produce multiple antimicrobial compounds, including ribosome-synthesized peptides, such as bacteriocin, and non-ribosome-synthesized peptides containing iturins, surfactins, and fengycins (Romero et al., 2007). Several *Bacillus* strains producing these metabolites are highly protective against phytopathogens. For example, *B. halotolerans* KLBC XJ-5 demonstrates excellent antifungal activity and contains six antimicrobial biosynthesis gene clusters (Wang et al., 2021). Potential biocontrol agents *B. velezensis* F85 and *B. amyloliquefaciens* T113 contain 49 and 51 genes involved in the biosynthesis of antibiotic compounds in their genome, respectively (Zhu et al., 2020). The genome of endophyte *B. halotolerans* strain BFOA4 harbors at least four biosynthetic gene clusters, which can be associated with its antifungal activity against *Fusarium* (Slama Ben et al., 2019). In this study, genome sequencing demonstrated that the *Bacillus* K1 contains 11 potential biosynthetic gene clusters encoding subtilosin, surfactin, bacillaene, fengycin, bacillibactin, and bacilysin. Notably, clusters 1, 5, 9, 10, and 11 showed a 100% similarity with known structures. Hence, it was suggested that K1 could produce a variety of antibiotics that be associated with its antagonistic activity against gray mold on grape fruit. However, in gene biosynthesis prediction, clusters 2, 3, 6, 7, and 8 with low similarity or unknown function suggested that *Bacillus* K1 could produce other unknown secondary metabolites. Meanwhile, 160 genes were clustered into the unknown function category in COG annotation, which further indicated that these genes might have novel functions. Future studies should focus on the investigation of these genes.

Endophytes are considered as beneficial microorganisms for their host (Bolívar-Anillo et al., 2019). They can modulate the plant immune system or inhibit pathogens by producing active substances, thus promoting plant health (Santoyo et al., 2016; Haidar et al., 2017). In this study, after inoculation, K1 suspension, supernatant, and fermentation broth all obviously decreased the disease incidence and decay extent of grapes and tomatoes. Remarkably, the K1 suspension treatment group showed the lowest incidence rate as compared to other treatments, which indicated that K1 endophytes promoted active interaction with pathogens in plant tissues. Similar results have been observed in *B. amyloliquefaciens* B4 against *Penicillium expansum* on loquat fruit (Ye et al., 2021).

Accumulated evidence has suggested that inoculation of beneficial microbes can act as elicitors to trigger the plant immune system (Saijo et al., 2018). Additionally, antagonistic microbe metabolites can induce disease resistance in plants (de Lamo and Takken, 2020).

The K1 culture could induce fruit defense reaction and improve disease resistance. The improved system defense depressed the growth of *B. cinerea* mycelia and decreased the disease incidence. The mechanism might be associated with the enhanced activity of defense-related enzymes. PAL is a key enzyme in the phenylpropanoid metabolic pathway, which decides the total phenol contents in living organisms (Barros et al., 2016). The change in PAL level was closely correlated with the content of disease-resistant substances. POD is a bifunctional enzyme. On the one hand, it plays an important role in the process of phenol polymerization and lignin synthesis, and on the other hand, it is related to plant resistance (Liebthal et al., 2018). POD can significantly suppress spore germination and hypha elongation of *Pseudocercospora abelmoschi, P. cruenta, Hibiscus esculentus*, and *Vigna sinensis* ssp. *sesquipedalis* in incubation experiment (Joseph et al., 1998). PPO can oxidate phenolics to more toxic quinines, which can fight the invading pathogens and are also related to lignin synthesis (Mohammadi and Kazemi, 2002).

In this study, K1 significantly elevated PAL, PPO, and POD activities in treatment fruit groups. Therefore, high levels of enzymes may be a key factor in reducing plant susceptibility to pathogens in grapes. A recent report showed that the activities of these defense-related enzymes in loquat fruit obviously are maintained at higher levels after inoculation with antifungal strain *B. amyloliquefaciens* B4 culture (Ye et al., 2021). In another study, antagonistic yeast *Pichia membranifaciens* reduces *Rhizopus* rot on peaches by enhancing the activities of PAL, PPO, and POD (Zhang et al., 2020).

After treatment with K1 suspension, the surface of grape fruit tissue was smooth and the pulp tissue was compact. In contrast, all fruits rotted in the positive control group, making the surface relatively softer and covered with a mold layer. As shown in Figure 9, numerous bacteria gathered on grape tissues and adhered to the hyphal surface. Thus, K1 endophytes could colonize and engage in complex interactions with pathogens.

The possible interaction between microorganisms was antibiosis. It was supported that the mycelia showed irregular depressions and pores at the end of inoculation (Figure 9D). In antimicrobial *Bacillus*, lipopeptides were the main antagonistic factors, since they could easily interact with lipid bilayers of pathogens' biological membranes and contributed to irreversible pore formation and further complete disruption and solubilization of the lipid bilayer (Ongena and Jacques, 2008; Fira et al., 2018). Secretion of a certain concentration of active lipopeptides in grape fruit might play an important role in the illumination mechanism attributing to the antibiosis between K1 and *B. cinerea*.

## Conclusion

In the study, we identified a wild grape endophytic *B. subtilis* K1 exhibiting strong antagonistic ability against *B. cinerea*. The strain could produce bioactive VOCs, thus, inhibiting the growth of pathogen mycelia. Among the 33 compounds identified *via* GC–MS in K1 fermentation, dibutyl phthalate was the main ingredient with a high content of 74.28%. It obviously decreased the disease incidence and decay degree of grape fruit by inducing the activities of defense-related enzymes and antibiosis. Bioinformatics analysis of the genome revealed that *Bacillus* K1 harbored 11 conserved biosynthesis gene clusters encoding subtilosin, surfactin, bacillaene, fengycin, and bacillibactin. SEM images showing an active interaction between K1 and fungi deduced that colonization and antibiosis are probably the mechanisms attributing to the high antifungal activity of K1. These results implied that *Bacillus* K1 might be developed as a potential biocontrol strain for grape storage. However, future studies should optimize fermentation conditions, explore the antagonistic activity of secondary metabolites, and investigate strain amplification during fruit storage. Moreover, the safety of K1 secondary metabolites for food safety should be evaluated.

## Data Availability Statement

The datasets presented in this study can be found in online repositories. The names of the repository/repositories and accession number(s) can be found in the article/Supplementary Material.

## Author Contributions

PL performed the experiments and reviewed the manuscript. BF wrote and reviewed the manuscript and provided financial support. ZY, BW, and YZ contributed to the data analysis. SS provided the plant materials. All authors approved the submitted version.

## Funding

This work was supported by the Natural Science Foundation of Shanxi (20210302123084 and 20210302123087), the Science and Technology Innovation Project of the Universities of Shanxi (2021l468), the Science and Technology Program of Yuncheng (YCKJ-2021029 and YCKJ-2021030), and Scientific Research Project of Characteristic Agricultural Product Development Group (SKX-202212).

## Conflict of Interest

The authors declare that the research was conducted in the absence of any commercial or financial relationships that could be construed as a potential conflict of interest.

## Publisher's Note

All claims expressed in this article are solely those of the authors and do not necessarily represent those of their affiliated organizations, or those of the publisher, the editors and the reviewers. Any product that may be evaluated in this article, or claim that may be made by its manufacturer, is not guaranteed or endorsed by the publisher.
