# Supplementary material for: Antifungal Activity of Endophytic Bacillus K1 Against Botrytis cinerea
Source: Front Microbiol. 2022 Jul 22;13:935675. doi: 10.3389/fmicb.2022.935675 (PMC9355035; doi:10.3389/fmicb.2022.935675)
Supplement: Supplementary file 1 [file Data_Sheet_1.docx]

**Antifungal activity of an endophytic *Bacillus* K1 against *Botrytis cinerea***

**Peiqian Li^1^, Baozhen Feng^1*^, Zhen Yao^1^, Bohui Wei^1^, Yanfei Zhao^1^, Shouguo Shi^1^**

^1^Key Laboratory of Plant Disease and Pest Control, Life and Science Department, Yuncheng University, Yuncheng, China


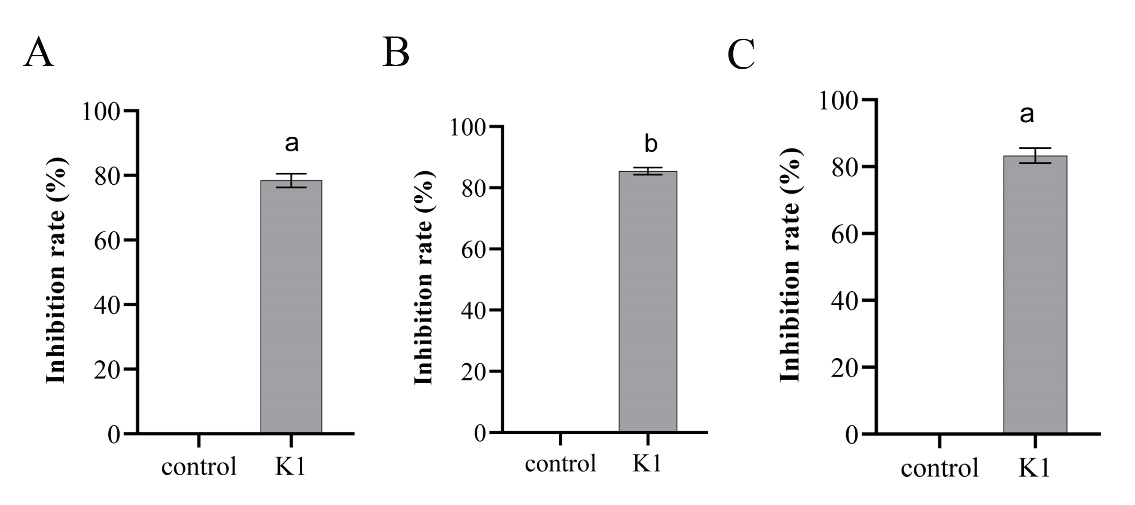


**Figure S1** **Antifungal evaluation of endophytic strain K1 against *Botrytis cinerea*.**

A, Quantitative analysis of antifungal activities of strain K1 against *B. cinerea*. B, Quantitative analysis of antifungal activities of K1culture extracts against *B. cinerea*. C, Quantitative analysis of antifungal activities of VOCs released by strain K1 against *B. cinerea*. Different lowercase letters indicated a significant difference (LSD’s multiple range test, p < 0.05).


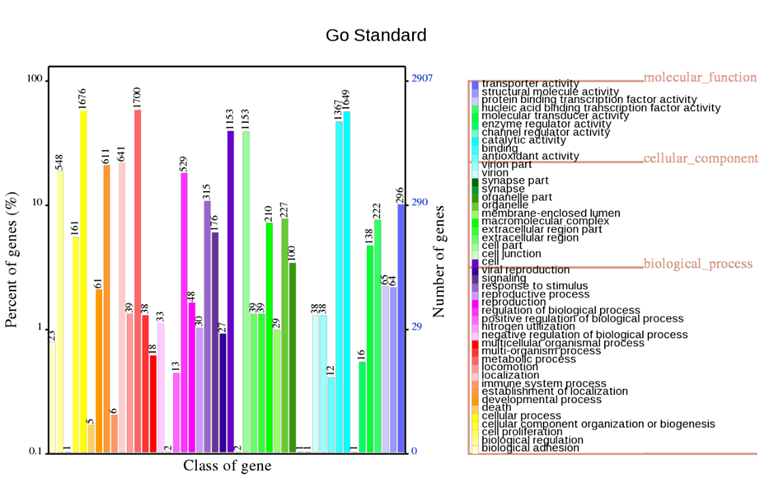


**Figure S2 GO annotation of *Bacillus* K1 genome.**


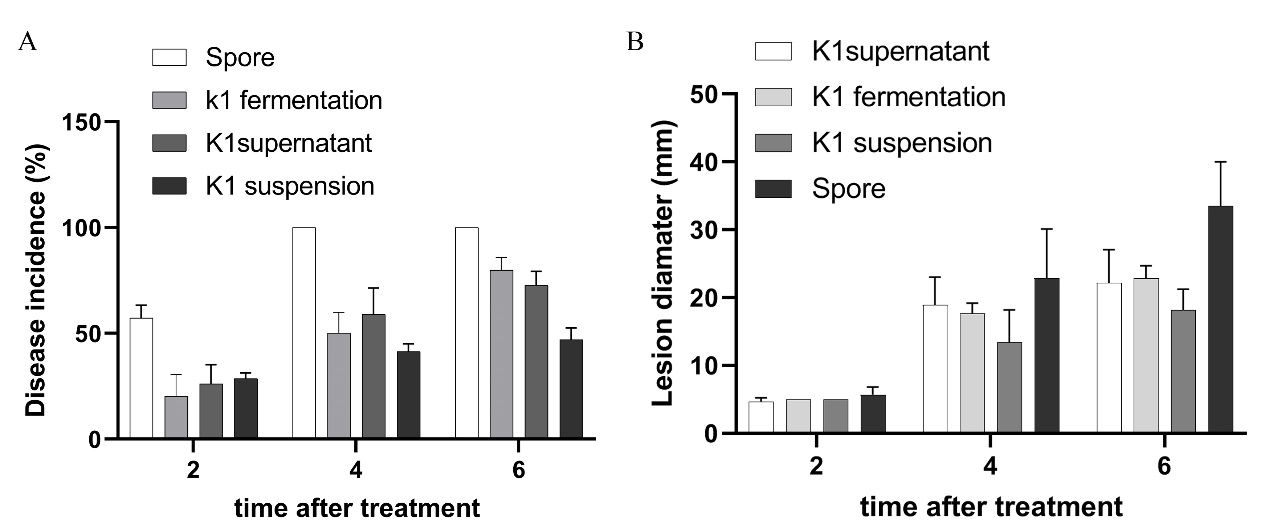


**Figure S3 Disease incidence and lesion diameter of cherry tomatoes after inoculation.**

A, Disease incidence on cherry tomato fruit after inoculation with *B. cinerea* and different K1 constituents. B, lesion diameters on diseased cherry tomato fruit after inoculation with *B. cinerea* and different K1 constituents. Three independent replicates were conducted for each treatment, 10 cherry tomatoes were used for each replication. The experiment was conducted thrice. Values are means of three replicates. Error bars represent standard error of the mean.

Table S1 Identification of VOCs of the *Bacillus* K1 by GC-MS

| Compounds name | Retention time (min) | Height | Area | Area% | CAS |
| --- | --- | --- | --- | --- | --- |
| Acetic acid, butyl ester | 2.709 | 189,437 | 3,418,832 | 0.164 | 000123-86-4 |
| Butanoic acid, 3-methyl- | 3.132 | 458,849 | 28,784,771 | 1.382 | 000503-74-2 |
| Butanoic acid, 2-methyl- | 3.278 | 904,510 | 47,121,621 | 2.2625 | 000116-53-0 |
| Pyrazine, 2,5-dimethyl- | 4.233 | 792,037 | 14,475,054 | 0.695 | 000123-32-0 |
| 1,3-Dioxolane-4-methanol, 2,2-dimethyl-, (S)- | 4.379 | 147,143 | 2,737,882 | 0.132 | 022323-82-6 |
| Hexanoic acid | 4.539 | 107,188 | 3,266,604 | 0.157 | 000142-62-1 |
| 1-Hexanol, 2-ethyl- | 4.646 | 351,446 | 6,379,552 | 0.306 | 000104-76-7 |
| 1-Octanol | 5.0943 | 143,690 | 3,911,473 | 0.188 | 000111-87-5 |
| Octanoic Acid | 6.229 | 329,926 | 7,544,862 | 0.3623 | 000124-07-2 |
| Tetradecane | 6.862 | 161,472 | 2,632,162 | 0.126 | 000629-59-4 |
| n-Decanoic acid | 7.592 | 130,464 | 3,393,606 | 0.163 | 000334-48-5 |
| Hexadecane | 8.181 | 118115 | 6,081,545 | 0.292 | 000544-76-3 |
| o-Acetylphenetidine | 10.299 | 293,483 | 19,382,244 | 0.931 | 000581-08-8 |
| Benzene, 1,4-dimethoxy-2,3,5,6-tetramethyl- | 10.538 | 259,776 | 15,946,169 | 0.766 | 013199-54-7 |
| 1,2,5,5,6,7-Hexamethylbicyclo[4.1.0]hept-2-en-4-one | 11.735 | 100,190 | 4,859,739 | 0.233 | 1000110-52-5 |
| Hexadecanoic acid, methyl ester | 12.909 | 170,182 | 10,976,802 | 0.527 | 000112-39-0 |
| Hexadecanoic acid, ethyl ester | 14.116 | 99,807 | 4,070,072 | 0.1954 | 000628-97-7 |
| 2-Cyclohexen-1-one, 4,4,5-trimethoxy- | 15.708 | 218,632 | 13,995,114 | 0.672 | 056180-51-9 |
| Hydrouracil, 1-methyl- | 16.102 | 260,661 | 11,578,131 | 0.556 | 000696-11-7 |
| L-Prolinamide | 16.735 | 128,190 | 5,216,380 | 0.251 | 007531-52-4 |
| Dibutyl phthalate | 17.787 | 16,173,026 | 1,547,120,613 | 74.282 | 000084-74-2 |
| Phenol, 3,5-dimethoxy- | 18.103 | 1,915,821 | 92,293,878 | 4.431 | 000500-99-2 |
| Dodecanamide | 18.3711 | 207,726 | 12,869,982 | 0.618 | 001120-16-7 |
| Phenol, 3,5-dimethoxy- | 18.770 | 172,987 | 12,000,622 | 0.576 | 000500-99-2 |
| 5-Chlorovaleric acid, morpholide | 19.014 | 113,990 | 3,860,847 | 0.185 | 1000307-35-6 |
| 2,3-Dimethyl-4-nitro-1-pyrrolidin-1-yl-butan-1-one | 19.632 | 207,890 | 7,960,917 | 0.382 | 1000194-90-5 |
| l-Leucine, N-cyclopropylcarbonyl-, hexadecyl ester | 19.827 | 1,440,112 | 74,961,756 | 3.600 | 1000327-78-4 |
| Pyrrolo[1,2-a]pyrazine-1,4-dione, hexahydro-3-(2-methylpropyl)- | 19.983 | 1,057,526 | 38,915,430 | 1.869 | 005654-86-4 |
| Bicyclo[2.2.2]octane-1-carboxylic acid | 20.348 | 128,593 | 4,195,874 | 0.202 | 000699-55-8 |
| l-Proline, N-allyloxycarbonyl-, heptadecyl ester | 20.591 | 187142 | 9,242,470 | 0.444 | 1000313-66-3 |
| Hexadecanamide | 20.840 | 106,073 | 4,638,063 | 0.223 | 000629-54-9 |
| 4-Octene, 2,3,7-trimethyl-, [S-(E)]- | 23.907 | 138,396 | 24,356,410 | 1.169 | 052763-13-0 |
| 9-Octadecenamide, (Z)- | 24.593 | 530,555 | 34,573,353 | 1.66 | 000301-02-0 |
